# Bariatric surgery in Poland, 2023: growth, trends, and impact of the KOS‑BAR program

**DOI:** 10.20452/wiitm.2024.17913

**Published:** 2024-11-19

**Authors:** Michał R. Janik, Przemysław Sroczyński, Piotr Major

**Affiliations:** General Surgery Department, Military Institute of Aviation Medicine, Warszawa, Poland; Second Department of General Surgery, Jagiellonian University Medical College, Kraków, Poland

**Keywords:** bariatric surgery, KOS‑BAR program, obesity, Poland, sleeve gastrectomy

## Abstract

**INTRODUCTION:**

Bariatric surgery is an effective treatment for obesity and metabolic disorders. In Poland, the growing prevalence of obesity has led to an increase in the demand for these procedures. Sleeve gastrectomy (SG) and Roux-en-Y gastric bypass (RYGB) are the most frequently performed surgeries, while newer techniques, such as single-anastomosis duodeno-ileal bypass with sleeve gastrectomy (SADI-S), single-anastomosis sleeve ileal bypass (SASI), and Nissen sleeve gastrectomy (N-SG) are emerging. The Comprehensive Specialist Care in Bariatrics (KOS-BAR) program, introduced in 2021, aims to standardize bariatric care in Poland.

**AIM:**

This study evaluates the state of bariatric surgery in Poland in 2023, focusing on procedure volumes, regional variations, and the role of KOS-BAR centers.

**MATERIALS AND METHODS:**

A nationwide survey was conducted among bariatric surgery centers affiliated with the Metabolic and Bariatric Surgery Chapter of the Association of Polish Surgeons. Data on the number and types of surgeries performed in 2023 were collected and compared with previous reports to analyze trends.

**RESULTS:**

A total of 54 centers, including 18 KOS-BAR centers, participated in the survey, reporting a total of 9102 procedures performed in 2023. SG accounted for 82% of surgeries, followed by RYGB (9.7%) and one anastomosis gastric bypass (3.9%). Newer procedures, such as N-SG (0.4%), SASI (0.8%), and SADI-S (0.2%) were less frequently performed. Over a half of all surgeries (59.3%) were performed in KOS-BAR centers. Significant regional variations in procedure volumes were observed.

**CONCLUSIONS:**

Bariatric surgery in Poland has seen substantial growth, with SG as the dominant procedure. However, regional disparities in access to care and the limited adoption of newer techniques persist. Expanding services to underserved regions and establishing a national registry are crucial for improving patient care and outcomes.

## INTRODUCTION 

Bariatric surgery has become a critical intervention in the management of obesity and related metabolic disorders, particularly for patients in whom conservative treatments were unsuccessful.^1^^; ^^2^^; ^^3^ In Poland, as in many other countries, the prevalence of obesity continues to rise, driving the increasing demand for bariatric procedures.^4^^; ^^5^ The most frequently performed procedures are sleeve gastrectomy (SG) and Roux-en-Y gastric bypass (RYGB), both of which have demonstrated efficacy in achieving significant and sustained weight loss.^6^^; ^^7^^; ^^8^^; ^^9^^; ^^10^^; ^^11^

In recent years, the field of bariatric surgery has evolved, with the introduction of newer techniques, such as single-anastomosis duodeno-ileal bypass with sleeve gastrectomy (SADI-S) and single-anastomosis sleeve ileal bypass (SASI).^12^^; ^^13^^; ^^14^^; ^^15^^; ^^16^ These procedures offer promising outcomes in terms of both weight loss and metabolic improvement, though they are still less widely adopted than the traditional surgeries, SG and RYGB.^17^ Analyzing the prevalence and adoption of these newer procedures, alongside more established techniques, is important to fully understand the current state of bariatric surgery in Poland.

To address the growing need for standardized and comprehensive bariatric care, the Comprehensive Specialist Care in Bariatrics (KOS-BAR) pilot program was introduced in Poland in 2021. It is a multidisciplinary comprehensive model of care for patients with pathological obesity, developed by the Polish Ministry of Health in cooperation with experts in bariatric surgery.^18^ The program is designed to ensure continuity of care, encompassing preoperative assessment, surgical treatment, and postoperative follow-up, thereby improving patient outcomes. A total of 19 centers participated in the KOS-BAR pilot program and received additional financial resources to support their operational activity.^19^ The surgical procedures are reimbursed separately from the lump sum. The source of the funding is the money obtained through the sugar tax imposed on products containing high levels of sugar, aimed at promoting healthier dietary choices and combating obesity.

While previous comprehensive reports on bariatric surgery in Poland were published in 2016 and 2019, significant progress in the field has been observed since then. In light of the introduction of the KOS-BAR program and increased adoption of newer surgical techniques, an updated analysis is warranted.

## AIM

The objective of this study was to evaluate the current state of bariatric surgery in Poland in 2023, with a particular focus on the distribution and activity of bariatric centers across the country. Using data collected through a nationwide survey, we aimed to analyze the volume and types of bariatric procedures performed in each voivodeship, as well as to assess the changes in surgery trends over time.

## MATERIALS AND METHODS

An online survey was carried out to collect data on the number and types of bariatric surgeries performed in 2023 as well as the characteristics of the bariatric centers. The questionnaire was distributed to all members of the Metabolic and Bariatric Surgery Chapter of the Association of Polish Surgeons. Follow-up reminders were sent via email and through personal contacts to the centers that did not respond initially. Inclusion in the survey was a prerequisite for being listed on the society’s official directory of institutions providing bariatric treatment, which is published on the society website. The survey results were compared to previous reports on bariatric surgery in Poland to analyze changes in surgery trends over time.^20^^; ^^21^^; ^^22^

### Statistical analysis

All statistical analyses were performed using SAS OnDemand for Academics package (SAS Institute Inc., Cary, North Carolina, United States). The study results were presented as percentages to enable clear visualization of variable distributions and facilitate comparative analysis across groups. This method ensures a precise representation of proportions, enhancing the interpretability of trends and differences.

### Ethics

As per institutional guidelines, this type of study did not require Institutional Review Board review or approval.

## RESULTS

A total of 54 bariatric surgery centers across Poland participated in the survey, including 18 designated as KOS-BAR centers. Overall, a total of 9102 bariatric procedures were performed across all centers in 2023 (Table 1). The most frequently performed surgery was SG, which accounted for 82% of all operations (7450 procedures). The second most common procedure was RYGB, making up 9.7% of all surgeries (883 cases). One anastomosis gastric bypass (OAGB) was performed in 3.9% of cases (356 procedures), while newer procedures, such as N-SG, SASI, and SADI-S were less frequently performed, constituting 0.4% (34 cases), 0.8% (76 cases), and 0.2% (19 cases) of all surgeries, respectively.

**Table 1:** Bariatric centers and operations by region in Poland in 2023

Region	Total centers, n	KOS‑BAR centers, n	Total number of bariatric procedures	SG, n (%)	RYGB, n (%)	N‑SG, n (%)	OAGB, n (%)	SASI, n (%)	SADI‑S, n (%)
Lower Silesian	7	2	1304	979 (75.1)	220 (16.9)	2 (0.2)	8 (0.6)	8 (0.6)	1 (0.1)
Kuyavian‑Pomeranian	3	3	1104	951 (86.1)	68 (6.2)	0	62 (5.6)	0	0
Lublin	3	1	428	354 (82.7)	64 (15)	0	1 (0.2)	2 (0.5)	0
Lubusz	2	0	205	159 (77.6)	7 (3.4)	5 (2.4)	33 (16.1)	6 (2.9)	0
Masovian	6	3	1079	948 (87.9)	59 (5.5)	0	9 (0.8)	28 (2.6)	0
Lesser Poland	3	1	763	697 (91.3)	57 (7.5)	0	0	3 (0.4)	5 (0.7)
Opole	1	0	119	84 (70.6)	34 (28.6)	0	0	1 (0.8)	0
Podlaskie	5	2	433	372 (85.9)	33 (7.6)	8 (1.8)	15 (3.5)	0	2 (0.5)
Pomeranian	6	3	1333	1068 (80.1)	120 (9)	0	118 (8.9)	4 (0.3)	10 (0.8)
Warmian‑Masurian	3	1	632	498 (78.8)	36 (5.7)	6 (0.9)	80 (12.7)	12 (1.9)	0
Greater Poland	2	0	184	145 (78.8)	36 (19.6)	5 (2.7)	0	3 (1.6)	0
West Pomeranian	1	0	336	275 (81.8)	61 (18.2)	0	0	0	0
Lodz	6	1	384	242 (63)	36 (9.4)	2 (0.5)	2 (0.5)	0	1 (0.3)
Silesian	5	1	739	628 (85)	49 (6.6)	6 (0.8)	22 (3)	9 (1.2)	0
Holy Cross	1	0	59	50 (84.7)	3 (5.1)	0	6 (10.2)	0	0
Total	54	18	9102	7450 (82)	883 (9.7)	34 (0.4)	356 (3.9)	76 (0.8)	19 (0.2)

Abbreviations: KOS‑BAR, Comprehensive Specialist Care in Bariatrics; NSG, Nissen sleeve gastrectomy; OAGB, one anastomosis gastric bypass; RYGB, Roux‑en‑Y gastric bypass; SADI‑S, single‑anastomosis duodenal‑ileal bypass with sleeve gastrectomy; SASI, single‑anastomosis sleeve ileal bypass; SG, sleeve gastrectomy

Regional variations in bariatric surgery volumes were observed, and these variations did not consistently correlate with population size. The Pomeranian voivodeship (population of 2 359 600) reported the highest number of procedures (n = 1333), followed closely by Lower Silesia (population of 2 879 300; n = 1304). Notably, in the Masovian voivodeship, despite it having the largest population (5 510 500), fewer surgeries were performed (n = 1079) than in regions with smaller populations. In Lesser Poland, with 3 429 600 inhabitants, 763 procedures were conducted. In contrast, the Holy Cross region (population of 1 168 500) and Opole (population of 936 700) reported markedly lower surgical volumes (n = 59 and n = 119, respectively).^23^ These disparities suggest that factors other than population size may influence bariatric surgery rates across different regions. Further research is needed to elucidate the specific determinants of these regional variations in bariatric surgery provision.

In all regions, SG consistently remained the most frequently performed procedure. For example, in the Masovian region, SG accounted for 87.9% of surgeries, and in the Pomeranian voivodeship, it made up 80.1% of the total. There were also notable differences in the frequency of alternative procedures. For instance, in Lesser Poland, no OAGB procedures were recorded, while in the Kuyavian-Pomeranian voivodeship, 5.6% of the procedures were OAGB. The frequency of newer procedures also varied across regions, with Podlaskie and Lubusz reporting 1.8% and 2.4% of their surgeries to be N-SG, respectively, whereas in many regions this procedure was not performed at all. Some regions reported minimal numbers of SASI or SADI-S procedures, indicating that these newer techniques are less commonly adopted at present.

KOS-BAR centers, which represented 33% of all bariatric centers participating in the survey, were responsible for a substantial portion (59.3%) of the surgeries performed, indicating their prominent role in providing bariatric care. Surgical distribution patterns did not differ markedly between the KOS-BAR and non–KOS-BAR centers, with SG being the predominant surgery across both categories. These findings illustrate a national trend favoring SG as the primary bariatric procedure, with only a minority of centers performing more complex procedures, such as RYGB, OAGB, N-SG, and SASI. The variation in procedure types across regions could be indicative of regional preferences, differences in patient profiles, or surgeon expertise.

In 2014, approximately 1499 bariatric procedures were performed, with the numbers increasing to 1958 in 2016.^20^^; ^^21^ By 2023, the number of procedures surged to 9102, reflecting a significant increase over the past decade. The percentage increase in bariatric surgeries in Poland between 2014 and 2023 is approximately 507%, while the increase between 2016 and 2023 is around 365%.

The trends in bariatric surgeries performed in Poland between 2007 and 2023 show significant changes in the distribution of various procedures. SG has emerged as the dominant procedure, with a dramatic increase in its use from 2009 to 2023. Initially comprising less than 16% of all procedures in 2009, SG surged to account for 82% by 2023, making it the most widely adopted bariatric surgery in Poland.

**Figure 1:**
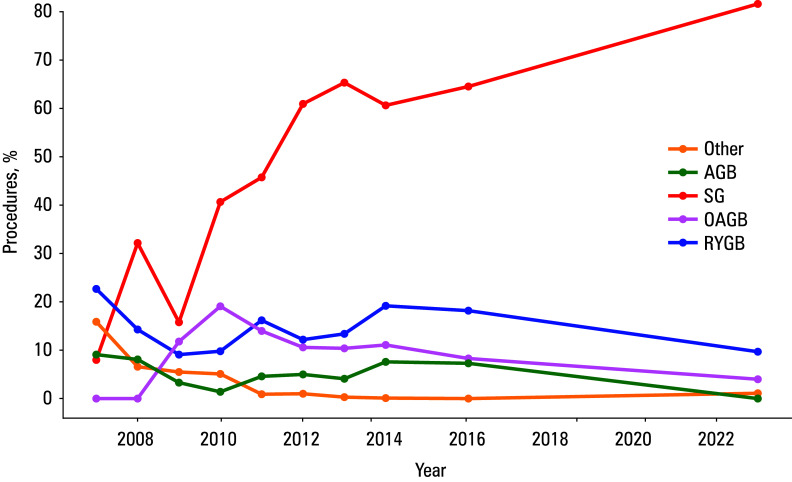
Trends in bariatric surgery in Poland (2007–2023)

Abbreviations: AGB, adjustable gastric banding; others, see Table 1

In contrast, the frequency of RYGB, once a leading procedure, declined steadily over the years, dropping from over 40% in 2007 to only 9.7% in 2023. Similarly, adjustable gastric banding, which made up 24% of all procedures in 2009, exhibited a sharp decline in frequency, becoming nearly obsolete by 2023. Newer procedures, such as OAGB, N-SG, SASI, and SADI-S, have been introduced more recently. OAGB gained some popularity, making up 3.9% of all procedures in 2023, while N-SG, SASI, and SADI-S, though still emerging, represented 0.4%, 0.8%, and 0.2% of all surgeries, respectively (Figure 1).

## DISCUSSION

The findings of this study offer an up-to-date overview of bariatric surgery in Poland in 2023. We observed a substantial increase in the number of bariatric surgeries performed in Poland between 2014 and 2023. This increase was likely driven by the rising obesity epidemic, increased awareness of the benefits of bariatric surgery, and the expansion of the KOS-BAR program, introduced in 2021 to standardize care and improve patient access to surgery. This rapid growth in Poland outpaces global trends. According to the International Federation for the Surgery of Obesity and Metabolic Disorders (IFSO) 2020–2021 survey,^24^ the total number of bariatric procedures worldwide increased from 507 806 in 2020 to 598 834 in 2021, marking an 18% increase. In the United States, the American Society for Metabolic and Bariatric Surgery (ASMBS) reported a 6.5% increase in the number of procedures performed from 2021 to 2022, rising from 262 893 to 280 000 surgeries.^25^ In Poland, however, the expansion has been much more pronounced. The growth in surgical volume over the past decade in Poland far exceeds the global average. This difference can be attributed to Poland’s evolving health care infrastructure and the strategic implementation of the KOS-BAR program, which has enhanced access to bariatric surgery and improved postoperative care.

When comparing the number and trends of bariatric procedures performed in Poland with global data, several parallels and differences emerge. As of 2023, Poland reported a total of 9102 bariatric surgeries, with SG being the most frequently performed procedure, accounting for 82% of all operations. This aligns with global trends reported by the IFSO and ASMBS, where SG remains the most performed bariatric procedure worldwide. According to the IFSO 2020–2021 survey,^24^ SG constituted 58% to 65.7% of all bariatric surgeries globally. In the European Chapter, SG accounted for 58% to 65% of all procedures in 2021. In the Asia-Pacific Chapter, SG made up 70% of all surgeries. In the Middle East and North African Chapter, SG represented over 80% of procedures, which is similar to the distribution observed in Poland. In North America, SG constituted 65.7% of procedures. Poland’s reliance on SG is closely aligned with the trends observed in the Middle East and North African countries, where the dominance of this procedure is similarly high, though it exceeds the proportions seen in the IFSO European Chapter and North America.^24^^; ^^25^

In Poland, RYGB made up 9.7% of all surgeries in 2023. This proportion is relatively low, as compared with other regions. In the IFSO European Chapter, RYGB accounted for about 22% to 26% of all bariatric procedures. In North America, the percentage of RYGB was around 22%.^24^^; ^^25^ In Latin America, the dominance of RYGB has decreased, and SG has emerged as the most frequently performed procedure. Thus, Poland’s reliance on RYGB is notably lower than what is observed in other regions, such as North America and the IFSO European Chapter, where this procedure is still more prevalent, though the global trend has been toward a reduction in RYGB volume in favor of SG.

Newer procedures, such as SADI-S and SASI, are still emerging both in Poland and worldwide. In Poland, these procedures accounted for a small fraction of all surgeries, and globally they also represent a minority of procedures. In 2022, SADI-S and similar procedures accounted for approximately 0.7% of all surgeries in the United States.^25^

Similarly, N-SG, a hybrid procedure, is also gaining traction but remains infrequently performed, accounting for only 0.4% of bariatric surgeries in Poland in 2023. While this procedure is not yet widely adopted, its potential benefits in addressing reflux issues post-SG may lead to its more widespread use in the future, both domestically and globally.^26^

A major factor shaping the current landscape of bariatric surgery in Poland is the introduction of the KOS-BAR program in 2021. Designed to provide comprehensive care for patients undergoing bariatric surgery, KOS-BAR has helped standardize care, improve outcomes, and ensure continuity of treatment from preoperative evaluation through postoperative follow-up. The data show that over a half of bariatric surgeries in Poland are performed in KOS-BAR centers. However, significant regional disparities persist. In some regions, such as Opole, Holy Cross, and West Pomeranian, there are no KOS-BAR centers, which may limit access to high-standard care in these areas.

Another key finding is the uneven distribution of bariatric surgery centers across Polish voivodeships. While regions such as Masovian, Lower Silesian, and Pomeranian have well-established bariatric centers, others, including Podkarpackie, are entirely unrepresented in the data. This lack of bariatric centers in certain regions reflects ongoing disparities in access to surgical care, which could be attributed to factors such as resource allocation, availability of trained surgeons, and regional demand for bariatric procedures. These disparities are concerning given the increasing prevalence of obesity across the country, and highlight the need for targeted efforts to expand bariatric services into underserved areas.

It is crucial to recognize that, despite the significant rise in the number of reported procedures, challenges in data collection persist. Previous reports have highlighted issues with inconsistent data submission from certain centers, and while some progress has been made, there is still a need for further improvements. The reliance on survey-based data collection poses additional limitations, as some centers may have provided inaccurate or incomplete information, and others may not have participated in the survey at all. To overcome these challenges and ensure more reliable data, creation of a national bariatric registry is essential. Such a registry would facilitate better resource planning and offer a more thorough understanding of patient outcomes and complications across different types of procedures.

## CONCLUSIONS

This study provided a detailed overview of bariatric surgery in Poland in 2023 and showed significant growth in the number of procedures, with a 507% increase since 2014, driven by the introduction of the KOS-BAR program. The analysis of 54 active centers, including 18 KOS-BAR centers, revealed substantial regional variations in surgical access and volume. While regions such as Pomerania and Lower Silesia were leaders in terms of the number of procedures performed (>1300 procedures each), others, for example, the Holy Cross voivodeship, reported minimal activity with only 59 procedures performed. SG remains the dominant procedure, accounting for 82% of all operations, while newer techniques, such as SADI-S, SASI, and N-SG, show limited adoption. Despite this progress, regional disparities in access to bariatric care persist, with some regions lacking bariatric centers or KOS-BAR participation. Additionally, the uneven distribution of newer procedures highlights differences in surgeon expertise and resource availability across regions. To address these challenges, expansion of services to underserved areas and improvements in data collection through creation of a national registry are crucial for enhancing patient outcomes and ensuring equitable access to bariatric care throughout Poland.
